# Evaluation of Systemic Antifungal Use in a Latin American General Care Hospital: A Retrospective Study

**DOI:** 10.3390/pharmacy11040108

**Published:** 2023-06-24

**Authors:** Abigail Fallas-Mora, Jose Pablo Díaz-Madriz, Jose Miguel Chaverri-Fernández, Esteban Zavaleta-Monestel

**Affiliations:** 1 Pharmacy Department, Hospital Clinica Biblica, San Jose 1307-1000, Costa Rica; afallasmo@clinicabiblica.com (A.F.-M.); jdiazm@clinicabiblica.com (J.P.D.-M.); 2 Department of Pharmacology, Toxicology and Pharmacodependence, University of Costa Rica, San Jose 1260-1000, Costa Rica; jose.chaverri@ucr.ac.cr

**Keywords:** antifungal agents, drug resistance, antimicrobial stewardship, Costa Rica

## Abstract

Background: Invasive fungal infections significantly contribute to mortality and morbidity rates. Despite the presence of all four major classes of antifungal medications, it is estimated that these infections result in the death of 1.5 million people each year, and death rates are increasing at an alarming rate. With increasing concerns about the emergence of antifungal resistance, there is a growing consideration in many countries to incorporate antifungal stewardship into existing antimicrobial stewardship programs. This approach aims to address issues hindering the appropriate use of antifungal drugs and to optimize their utilization. Methods: An analytical retrospective study of 48 hospitalized patients was conducted to assess factors related to the use of systemic antifungals and develop and implement an internal protocol to improve its use. Results: All patients with severe comorbidity had SOFA scores linked with a mortality risk of more than 10%. Based on 48 evaluations of antifungal orders, 62.5% were considered appropriate, 14.6% were considered debatable, and 22.9% were considered inappropriate. Infectious disease physicians made most of the prescriptions considered appropriate in this study. Conclusions: Comorbidities and risk factors in patients receiving systemic antifungals can be associated with the development of more serious fungal infections; hence, the implementation of antifungal stewardship as a complement to antimicrobial stewardship programs can help facilitate decision-making when dealing with a suspected case of fungal infection.

## 1. Introduction

The prevalence of fungal infections has significantly increased over time, especially those associated with healthcare. The discovery and use of increasingly invasive techniques for the treatment of complicated diseases, such as central venous catheters, is primarily responsible for this trend. Additionally, due to their vulnerability, there has been an increase in patients with immunological impairment linked to pharmaceutical therapy. Given the high mortality rate associated with delayed treatment, fungal infections are considered a severe issue in the current hospital context [1,2].

Invasive fungal infections significantly contribute to mortality and morbidity rates. Despite the availability of four major classes of antifungal medications, it is estimated that these infections result in the death of 1.5 million people each year, and death rates are increasing at an alarming rate [3]. The incidence of mucosal and systemic mycoses continues to rise due to the use of broad-spectrum antibiotics, chemotherapy, immunosuppressive regimens required for solid organ transplantation, as well as the ongoing HIV and type 2 diabetes pandemics [2].

The COVID-19 pandemic has led to a significant increase in the use of antibiotics and antifungal drugs in hospitals worldwide. This rise in antifungal drug usage is primarily attributed to the emergence of COVID-associated pulmonary aspergillosis, COVID-associated mucormycosis, and COVID-associated invasive candidiasis, which have complicated the clinical course of the disease [4]. It is also believed that the COVID-19 pandemic may have exacerbated antifungal resistance due to the heightened utilization of antifungal drugs in hospitals and other healthcare settings [3,5].

Among the various fungal species causing systemic fungal infections, *Candida* spp. is particularly notable, accounting for approximately 80% of nosocomial fungal infections. In Costa Rica, the use of central venous catheters and parenteral nutrition has been associated with a high incidence of *Candida parapsilosis* infections, especially in public hospitals. Additionally, in settings where infrastructure development or repair processes are underway, such as in our case, there is a notable increase in *Aspergillus* spp. infections within the hospital environment [1,6].

One critical issue associated with these fungal infections is the high prevalence of azole resistance, which poses a challenge for antifungal treatment. Resistance occurs when therapeutic concentrations of a specific antifungal fail to inhibit the growth of fungal colonies or when extremely high serum concentrations of the medication are required to eliminate the microorganism, posing significant risks to the patient. Fungi can develop various mechanisms of resistance, including biofilm formation, enhanced drug efflux, and genomic alterations that reduce their sensitivity to antifungal drug toxicity [6].

Identifying candidemia and other invasive fungal infections is complicated by the presence of resistance and the need to consider multiple risk factors for each patient. Furthermore, a direct tool for assessing the severity and prognosis of patients with candidemia is currently unavailable [7]. However, the use of risk scores in the treatment of candidemia has gained increasing interest. Through evaluating the patient’s risk, it becomes possible to promptly initiate treatment in those who require it while avoiding unnecessary antifungal use in patients who are not at risk of developing candidemia [7,8].

Several scoring systems are utilized to assess the risk of candidemia in both ICU and non-ICU patients, including APACHE II, the Sequential Organ Failure Assessment (SOFA) score in adults, and the qSOFA. While APACHE II is a complex scoring system, studies have been conducted to validate simpler scores such as SOFA and the Charlson Comorbidity Index (CCI) for estimating candidiasis risk [4]. Additionally, specific scores such as the Invasive Fungal Infection Risk Prediction Score have been developed to directly estimate the risk of candidiasis in patients [9].

Globally, stewardship programs have been established to preserve the effectiveness of antimicrobials in treating infectious diseases. These programs aim to limit the development of resistance in harmful microorganisms, primarily focusing on antibiotics [4]. However, with increasing concerns about the emergence of antifungal resistance, there is a growing consideration in many countries to incorporate Antifungal Stewardship (AFS) into existing stewardship programs. This approach aims to address issues hindering the appropriate use of antifungal drugs and optimize their utilization [10,11].

Since pharmacists play many roles in AFS, including drug selection, dose and dosage, confirmation of treatment duration, and therapeutic drug monitoring, this study aims to assess the use of systemic antifungals at Hospital Clínica Biblica (HCB) and to determine whether comorbidities and risk factors in patients receiving systemic antifungals may be associated with the development of more serious fungal infections, in order to facilitate the implementation of a hospital policy that allows for the best possible use of these medications and, consequently, yields the best clinical results associated with its use [10].

## 2. Materials and Methods

### 2.1. Study Design and Settings

This is a retrospective observational study of patients who were treated in the Intensive Care Unit (ICU), Coronary Unit, and Intermediate Care Unit of HCB, a 78-bed private healthcare center located in San José, Costa Rica. The analysis was conducted between January and December of 2021.

### 2.2. Inclusion and Exclusion Criteria

The study identified patients hospitalized during the specified periods through consulting the hospital’s electronic medical records. All patients who received treatment with any of the systemic antifungals available in the hospital were included in the analysis. Patients under 18 years, those with a hospital stay of fewer than two days, individuals with a terminal illness or under palliative care, and those who had tested positive for COVID-19 were excluded from the study.

### 2.3. Data Collection

Starting in January 2021, a retrospective analysis was conducted on 77 patients who received systemic antifungal therapy. A data collection sheet was created to gather general patient characteristics, including age, gender, admission diagnosis, and length of stay in the hospital. The sheet also included the severity of underlying clinical conditions determined using the CCI (Table 1) and SOFA score in adults (Table 2), and the presence of invasive fungal infection risk factors was determined using the Invasive Fungal Infection Risk Prediction Score (Table 3).

Regarding the utilized scores, the original version of the CCI was developed to predict ten-year mortality based on 19 distinct underlying diseases and medical conditions. Each condition is assigned a score of 1, 2, 3, or 6 based on the risk of death, and the total score is calculated to predict mortality. The index includes a risk stratification that is divided into three categories: mild comorbidity (0–2 points), moderate comorbidity (3–4 points), and severe comorbidity (≥5 points) [7,9,12].

The SOFA score is a predictive tool for morbidity and mortality initially designed for use in the ICU. However, it is now widely utilized in non-ICU patients as well. This tool incorporates six criteria that assess the functioning of various body systems, including the respiratory, cardiovascular, renal, neurological, hepatic, and hematological system. Each criterion is assigned a score ranging from 1 to 4, as outlined in Table 2. Risk stratification is determined based on the total score: a score of 0 to 6 indicates a mortality risk of less than 10%, a score of 7 to 9 indicates a risk of 15–20%, a score from 10 to 12 corresponds to a risk of 40–50%, a score of 13 to 15 indicates a risk greater than 80%, and a score between 16 and 24 signifies a risk exceeding 90% [13,14].

On the other hand, the Invasive Fungal Infection Risk Prediction Score is divided into three categories: low risk (≤8 points), moderate risk (9–13 points), and high risk (≥14 points), with a maximum score of 26 points. A higher score indicates a greater risk of infection [9].

Information on the prescribed antifungals (including the route of administration, dose, and treatment duration) and cultures was collected and analyzed to determine whether the systemic antifungal order was consistent with local resistance patterns and international guidelines. The information regarding the culture results and strain susceptibilities was provided by the microbiologist from HCB, who is an integral member of the multidisciplinary infectious diseases team. Data on the need for and performance of dose adjustments based on renal clearance and hepatic insufficiency were also tabulated.

To evaluate the optimization of antifungal use, the criteria previously described by Nivoix et. al., modified for use by the HCB infectious diseases team, were utilized. These criteria included indication, dosage, and potential antifungal–drug interactions (as outlined in Table 4). The use of antifungals was considered appropriate when all three evaluation criteria were met, debatable when at least one debatable assessment criterion was present without any inappropriate assessment criteria, and inappropriate when at least one inappropriate assessment criterion was present [15,16].

Indications and dosages were assessed based on the recommendations provided by the Infectious Diseases Society of America (IDSA) guidelines for the diagnosis and management of aspergillosis and candidiasis, according to the recommendations of the clinical pharmacist and infectious disease physician from the multidisciplinary infectious diseases team at HCB. The potential for antifungal–drug interactions and their associated risk category was assessed using Lexi-Comp Inc. (Hudson, OH, USA) Online software [17,18].

### 2.4. Statistical Analysis

The data were entered, classified, and analyzed using Microsoft^®^ Excel^®^ for Microsoft 365 MSO (Microsoft, Redmond, Washington, DC, USA) and IBM^®^ SPSS Statistics version 28 (IBM, Chicago, IL, USA).

Descriptive statistics, such as frequencies and percentages, were calculated. The percentage of optimal prescriptions was determined based on international treatment guidelines and the protocol developed by Nivoix et al. [16].

### 2.5. Ethics Approval and Consent to Participate

Ethical approval was obtained from the Scientific Ethical Committee of the University of Costa Rica, Costa Rica (approval date 25 March 2019), approval reference number CEC-143-2019, to conduct the study. Written consent was not required as no direct interventions affecting the patients were performed.

## 3. Results

### 3.1. Patient Demographics and Comorbidities

Out of the 77 patients initially analyzed, only 48 met the inclusion criteria and were included in the final study population. Table 5 provides an overview of the demographic characteristics and comorbidities of these 48 patients, while Table 6 presents the SOFA score and the Invasive Fungal Infection Risk Prediction Score for each patient.

The average age of the final study population was 72 years, ranging from 29 to 102, with a standard deviation of ±19. Among the included patients, 47.9% were female. The average hospitalization time was 12 days, with a standard deviation of ±10. Patients received antifungal therapy for an average duration of 10 days, with a standard deviation of ±8.

With the CCI (Table 5), it was observed that 50% of the patients had a severe level of comorbidity. The most prevalent comorbidities included diabetes mellitus, cancer, and moderate to severe renal disease, among others.

According to the SOFA score data (Table 6), only 25% of the patients had a mortality risk greater than 10%. In addition, the risk of getting invasive fungal infections was high in 22.9% of the patients and moderate in 27.1%. The use of complete parenteral feeding, central venous catheter implantation, and broad-spectrum antibiotic treatment for more than four days were the clinical variables most related to the risk of invasive fungal infection.

It was found that all patients with severe comorbidity had SOFA scores linked with a mortality risk of more than 10%. This was regarding the correlation between the CCI and the SOFA score. In total, three patients (6.2%) had both a severe comorbidity score and a SOFA score greater than 40–50%, which resulted in death as outcome.

### 3.2. Antifungal Use

**Figure 1:**
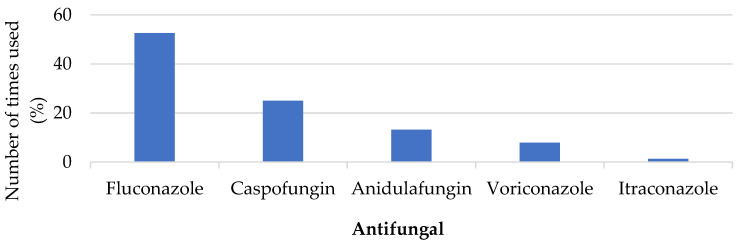
Distribution of antifungal use.

A total of 76 antifungals were used among the 48 patients, with fluconazole being the most widely used (52.6%), followed by caspofungin (25.0%) and anidulafungin (13.2%). These results are shown in Figure 1.

Out of the 48 patients, only 15 (31.2%) underwent at least one fungal culture, resulting in a total of 23 cultures. However, fungal species were detected in only 47.8% of the cultures. *Candida albicans* was the most frequently identified species, accounting for 63.6% of the positive cultures. Additionally, other non-albicans species, including *C. tropicalis* and *C. glabrata*, were also detected; these findings are presented in Table 7. Only 1 of the 23 cultures conducted revealed the presence of antifungal resistance, specifically fluconazole resistance exhibited by *Saccharomyces cerevisiae*. As a result, the infectious diseases specialist made the decision to switch the treatment to caspofungin.

**Figure 2:**
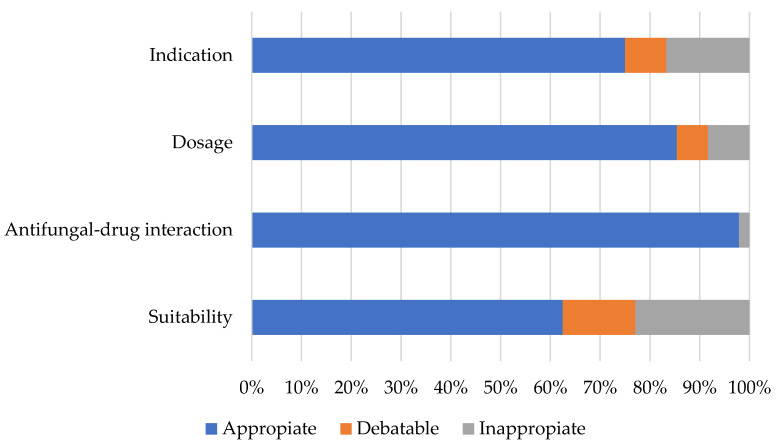
Suitability of the prescription of antifungals.

Regarding the evaluation of the use of antifungals, it was found that 62.5% of the prescriptions were appropriate according to the metric of Nivoix et al., 14.6% were considered debatable, and 22.9% were considered inappropriate [13,16]. These data can be found in Figure 2.

From the total number of prescriptions that were considered inappropriate, seven (58.3%) were considered an inappropriate indication, four (33.3%) were considered an inappropriate dose, and only one (8.3%) was considered inappropriate due to the presence of drug interactions. About the debatable prescriptions, four (57.1%) were considered a debatable indication, and three (42.9%) were considered a debatable dose.

## 4. Discussion

Since 2015, HCB has implemented the Antimicrobial Optimization Program (PROA), a stewardship program aimed at ensuring the appropriate use of antimicrobial drugs. However, the program has primarily focused on antibiotics and to a lesser extent on antifungals. The objective of this study was to analyze the systemic antifungal pharmacotherapy utilized in the hospital, with the aim of assessing the need for establishing new guidelines to minimize complications and fungal species resistance while ensuring the successful resolution of patient infections.

Multiple studies indicate that the mortality rate attributed to fungal infections rises in direct proportion to the delay in initiating appropriate therapy. Furthermore, current diagnostic methods, such as microbiological cultures, do not provide immediate results [1,19,20]. As a result, the utilization of risk scores for fungal infections has gained popularity. These scores enable the stratification of patients based on their morbidity and mortality risks and provide valuable information regarding the necessity of administering antifungal agents [9].

In this study, the CCI was utilized to assess the patients’ comorbidities [12]. The decision to employ this index as a determinant of comorbidities was based on two reasons: first, evidence suggests that despite the passage of time and medical advancements, the CCI remains a reliable and highly sensitive index, meeting current clinometric criteria; secondly, there is evidence indicating that the combined use of the CCI and the SOFA score may be a superior predictor of severity and prognosis in candidemia patients compared to the highly complex APACHE II index [7,12].

The study revealed that out of the 24 patients classified under the severe comorbidity group according to the CCI, only 3 patients presented a SOFA score with a risk of mortality exceeding 10%. Remarkably, all three of these patients died before their discharge, underscoring the significance of combining both indices when assessing the risk of mortality. Among the patients with severe CCI and a SOFA score below 10%, only one patient, diagnosed with healthcare-associated pneumonia, succumbed to their condition. These findings align with other studies that have associated higher SOFA scores with increased 30-day mortality [7,21].

The Invasive Fungal Infection Risk Prediction Score, developed to assess the likelihood of invasive fungal infections in ICU patients [9], was also employed in this study. Its validity has been established for hospitalized patients outside the ICU as well [22]. In this study, the score was used to justify the use of antifungal agents in patients without a clear indication for such treatment. Delaying antifungal administration in high-risk patients with a potential for invasive fungal infections can significantly impact patient outcomes [2].

Among the 33 patients who did not undergo culture confirmation of fungal presence at infection sites, appropriate prescription was deemed suitable in 21 cases. Out of these 33 patients, only 8 (24.2%) were classified as high-risk for invasive fungal infection. However, due to the severity of the infection and the associated risk of mortality (which exceeded 40% in five patients), the prescription’s appropriateness was considered justified in all instances. In the remaining three patients, the prescription was deemed appropriate due to suspected infection at a catheter insertion site in one case and the presence of neutropenia in the other two cases. The IDSA guidelines for candidiasis management state that empirical antifungal therapy should be promptly initiated in critically ill patients with risk factors for invasive candidiasis, along with the presence of other risk factors, surrogate markers for invasive candidiasis, and/or culture data [17].

Of the remaining 25 patients who did not undergo a culture, 10 were classified as being at moderate risk of invasive fungal infection, while 15 were classified as being at low risk. Among the ten patients with moderate risk, the appropriateness of the prescription was considered on eight occasions. In six cases, this was due to the presence of gastrointestinal perforation, and in the other two cases, it was attributed to severe oropharyngeal candidiasis, which aligns with the guidelines and supporting evidence [17,23]. On the other hand, for the patients at low risk, the prescription was considered appropriate in five cases due to the presence of neutropenia, septic shock, bacteremia of unknown origin, and severe oropharyngeal candidiasis, in accordance with the IDSA guidelines [17].

The appropriateness of the prescription for the remaining 10 patients without culture was considered debatable in 3 cases and inappropriate in 7 cases. In the debatable cases, this was due to a dosage that did not align with the recommended treatment guidelines, although the extent of deviation did not warrant deeming it inappropriate. However, in the inappropriate cases, it was deemed so on five occasions due to the indication itself and on two occasions due to the dosage utilized, as per the Nivoix et al. protocol [16,17].

Regarding the indication, it was deemed unnecessary to prescribe antifungals in these patients due to the absence of risk factors. Evidence suggests that such prescriptions only contribute to the potential development of antifungal resistance [15,24,25,26]. Regarding the dosing, adjustments should have been made to the fluconazole dose in two patients due to impaired renal function. In these cases, the dose was considered inappropriate due to the potential risk of fluconazole accumulation and its impact on liver function [27,28].

Out of the 15 patients who underwent fungal culture, the prescription adequacy was deemed appropriate in 10 cases, as the prescribed antifungals provided adequate coverage and doses for the identified fungal infections. As for the remaining five cases, the appropriateness of the prescription was considered debatable in two patients who were at low risk of invasive fungal infection, which contradicts clinical guidelines. However, some sources suggest that adding an antifungal in patients who show no clinical improvement after receiving broad-spectrum antibiotics may be justified; thus, the indication was not considered inappropriate in these cases [29].

Regarding the presence of resistance in the fungal isolates, only the positive culture for *S. cerevisiae* showed resistance to fluconazole, which is part of the intrinsic resistance that yeasts have to this antifungal agent [30,31]. For the other 22 cultures, all species showed susceptibility to the antifungals available in the hospital, indicating a positive indication of the current resistance profile. Further studies are needed to confirm the local susceptibility to antifungals at HCB.

Concerning the cases with cultures considered inappropriate, two were due to the indication, and one was due to the dosage. Regarding the first two, in one case, an antifungal was prescribed to a patient with *C. albicans* in a respiratory tract test, despite the patient having no comorbidities or risk factors for invasive fungal infection. According to the guidelines, this is considered colonization and does not require antifungal treatment [17,32]. The other case involves a patient without risk factors who showed clinical improvement in pneumonia and decided to initiate the antifungal treatment as a supplement to therapy. The case considered inappropriate in terms of dosage was a patient with renal dysfunction who required dose adjustment, but the prescribed dose exceeded the recommended adjustment by more than 25% [28].

It is important to consider that the majority of prescriptions considered appropriate in this study were made by infectious disease specialists, and none of the inappropriate prescriptions were made by these specialists. This highlights the significance of consulting infectious disease specialists in complex infections, as it has been observed that this is the most effective way to tailor antimicrobial therapy to the specific needs of each case without taking unnecessary risks [33]. Additionally, since a significant number of debatable and inappropriate prescriptions were related to antifungal dosing, this presents an opportunity to provide education to healthcare practitioners and further involve pharmacists in the multidisciplinary infectious disease team [10].

The main limitation of this research is the limited availability of data from fungal species cultures. Unlike bacterial cultures, not all patients receiving antifungal treatment at HCB undergo culture monitoring, which is recommended according to clinical guidelines. Additionally, there are cases where a susceptibility report for fungal species is not obtained from the laboratory along with the cultures, thereby impeding the appropriate de-escalation of therapy when necessary.

Moreover, the hospital currently lacks clinical guidelines for the treatment or prophylaxis of fungal infections, resulting in a scarcity of local information sources to assist physicians in making treatment decisions. Consultation with infectious diseases specialists is solely dependent on the individual physician’s discretion.

The findings of this study confirm the initial suspicion that the implementation of an Antifungal Stewardship Program within the stewardship program of HCB is necessary. Such a program would promote the appropriate use of antifungals in the hospital, involving not only physicians but also nursing staff, pharmacists, and other healthcare professionals involved in the process. Numerous studies demonstrate that the integration of other healthcare professionals, such as pharmacists, into stewardship programs not only enables them to contribute to the development of local prescription guidelines but also allows for broad collaboration in educational initiatives for the implementation of these guidelines. Pharmacists can play a crucial role in conducting continuing education programs for healthcare providers and nurses as well as patient education [34,35].

Moreover, pharmacists can assist in monitoring antimicrobial prescription rates, providing valuable data for interventions by the hospital’s infectious diseases team. Clinical pharmacists, who possess expertise in infectious diseases, can conduct antimicrobial regimen reviews to intervene in areas such as antifungal discontinuation, duration, de-escalation, and optimization of dose or frequency [35]. The implementation of Antifungal Stewardship Programs provides an opportunity for these professionals to actively collaborate in the patient treatment process.

## 5. Conclusions

For the patients included in the study, it was possible to collect clinical and laboratory parameters to assess the use of systemic antifungals at HCB and determine that comorbidities and risk factors in patients receiving systemic antifungals can be associated with the development of more serious infections. The CCI, the SOFA score, and the Invasive Fungal Infection Risk Prediction Score are valuable tools for assessing comorbidities and predicting the likelihood and prognosis of invasive fungal infections in patients receiving systemic antifungals.

Finally, the study emphasizes the importance of optimizing the use of systemic antifungals at HCB through the implementation of an Antifungal Stewardship Program, involving collaboration among healthcare professionals, education initiatives, and evidence-based prescribing guidelines. This approach can lead to better clinical outcomes and mitigate the risks associated with invasive fungal infections.

## Figures and Tables

**Table 1:** Charlson Comorbidity Index.

Risk Factor	Score	Risk Factor	Score
Miocardial infarction	1	Diabetes with end organ damage	2
Peripheral vascular disease	1	Moderate or severe renal disease	2
Cerebrovascular disease	1	Hemiplegia	2
Congestive heart failure	1	Any tumor without metastasis	2
Peptic ulcer disease	1	Leukemia	2
Diabetes	1	Lymphoma	2
Chronic pulmonary disease	1	Moderate or severe liver disease	3
Connective tissue disease	1	AIDS	6
Dementia	1	Metastatic solid tumor	6
Mild liver disease	1		

**Table 2:** Sequential Organ Failure Assessment (SOFA) score in adults.

System	Score				
	0	1	2	3	4
Respiration PaO_2_/FiO_2_, mmHg	≥400	301–399	201–300	101–200	≤100
Coagulation Platelets, ×10^3^ μL^−1^	>150	101–150	51–100	21–50	≤20
Liver Bilirubin, mg dL^−1^	<1.2	1.2–1.9	2.0–5.9	6.0–11.9	>12.0
Cardiovascular Mean Arterial Pressure, mmHg	Hypotension absent	<70	On dopamine ≤ 5 mcg/kg/min or any dobutamine	On dopamine > 5 mcg/kg/min, epinephrine ≤ 0.1 mcg/kg/min, or norepinephrine ≤ 0.1 mcg/kg/min	On dopamine > 15 mcg/kg/min, epinephrine > 0.1 mcg/kg/min, or norepinephrine > 0.1 mcg/kg/min
Central nervous system Glasgow coma scale	15	13–14	10–12	6–9	<6
Renal Creatinine, mg dL^−1^	<1.2	1.2–1.9	2.0–3.4	3.5–4.9	>5

**Table 3:** Invasive Fungal Infection Risk Prediction Score.

Risk Factor	Score
Diabetes	5
Gastrointestinal surgery	5
Hematologic malignancies	4
Broad-spectrum antibiotic therapy ≥ 4 days	4
Central venous catheter (CVC)	3
Total parenteral nutrition	3
Mechanical ventilation ≥ 2 days	2

**Table 4:** Criteria used to assess adherence to antifungal treatment guidelines.

Assessment	Indication	Dosage	Antifungal–Drug Interaction
Appropriate	Follows recommended practices by the infectious diseases team, local procedures, and/or published guidelines.	Appropriate dose x or underdose or overdose by ≤10% to loading dose when recommended. Also, observing the recommended dose limit and dose adjustments for renal dysfunction.	Antifungal has no potential interaction with drugs used concomitantly. Antifungal presents potential interactions with moderate severity but is subjected to clinical monitoring and dose adjustment when required.
Debatable	It does not follow protocol, but there is evidence in the literature or no suitable alternative.	Underdose or overdose x by ≤25% or no loading dose or no discontinuation or dose adjustment in case of clinically related adverse events.	Antifungal presents potential interactions with moderate severity, and clinical monitoring or dose adjustment is not performed when required.
Inappropriate	Inappropriate antifungal selection concerning the protocol or mycological data, despite the existence of a suitable alternative.	Under or overdose x > 25%; no discontinuation or dose adjustment in case of a clinically related adverse event when an appropriate alternative is available.	Antifungal presents potential interactions with concomitant medications, including severe or contraindicated interactions; the antifungal is used with concomitant drug therapy and results in failure of the antifungal, or there is concomitant use of two antifungals of the same classification.

**Table 5:** Demographics and comorbidities.

Characteristics	
Demographics	
Age (years), median (IQR)	72 ± 19
Male sex, *n* (%)	25 (52.1)
Outcome	
Success	37 (77.1)
Transfer to another facility	3 (6.2)
Death	8 (16.7)
Comorbidities	
Miocardial infarction, *n* (%)	3 (6.2)
Peripheral vascular disease, *n* (%)	0 (0.0)
Cerebrovascular disease, *n* (%)	2 (4.2)
Congestive heart failure, *n* (%)	5 (10.4)
Peptic ulcer disease, *n* (%)	8 (16.7)
Diabetes, *n* (%)	9 (18.8)
Chronic pulmonary disease, *n* (%)	7 (14.6)
Connective tissue disease, *n* (%)	0 (0.0)
Dementia, *n* (%)	4 (8.3)
Mild liver disease, *n* (%)	1 (2.1)
Diabetes with end organ damage	4 (8.3)
Moderate or severe renal disease, *n* (%)	8 (16.7)
Hemiplegia, *n* (%)	0 (0.0)
Any tumor without metastasis, *n* (%)	10 (20.8)
Leukemia, *n* (%)	1 (2.1)
Lymphoma, *n* (%)	3 (6.2)
Moderate or severe liver disease, *n* (%)	3 (6.2)
AIDS, *n* (%)	0 (0.0)
Metastatic solid tumor, *n* (%)	1 (2.1)
Charlson comorbidity index	
Mild comorbidity, *n* (%)	8 (16.7)
Moderate comorbidity, *n* (%)	16 (33.3)
Severe comorbidity, *n* (%)	24 (50.0)

Abbreviation: IQR, interquartile range.

**Table 6:** SOFA and Invasive Fungal Infection Risk.

Characteristics	
SOFA score	
0–6 points/mortality <10%, *n* (%)	36 (75.0)
7–9 points/mortality 15–20%, *n* (%)	4 (8.3)
10–12 points/mortality 40–50%, *n* (%)	6 (12.5)
13–14 points/mortality 50–60%, *n* (%)	1 (2.1)
15–24 points/mortality ≥90%, *n* (%)	1 (2.1)
Risk Factors for Invasive Fungal Infection	
Diabetes, *n* (%)	9 (18.8)
Gastrointestinal surgery, *n* (%)	12 (25.0)
Hematologic malignancies, *n* (%)	2 (4.2)
Broad-spectrum antibiotic therapy ≥4 days, *n* (%)	36 (75.0)
Central venous catheter (CVC), *n* (%)	26 (54.2)
Total parenteral nutrition, *n* (%)	13 (27.1)
Mechanical ventilation ≥2 days, *n* (%)	10 (20.1)
Invasive Fungal Infection Risk	
Low risk, *n* (%)	24 (50.0)
Moderate risk, *n* (%)	13 (27.1)
High risk, *n* (%)	11 (22.9)

**Table 7:** Results of the cultivation and determination of species.

Characteristics	
Patients with cultures for fungi, *n* (%)	15 (31.2)
Total of cultures for fungi, *n* (%)	23 (100)
Positive cultures for fungi, *n* (%)	11 (47.8)
Positive cultures according to each fungal species found	
*Candida albicans*, *n* (%)	7 (63.6)
*Candida tropicalis*, *n* (%)	3 (27.3)
*Candida glabrata*, *n* (%)	1 (9.1)
*Saccharomyces cerevisiae*, *n* (%)	1 (9.1)

## Data Availability

The data presented in this study are available on request from the corresponding author.
